# 

**Published:** 1872-12

**Authors:** 


### Contents of December Number.

### ORIGINAL DEPARTMENT.

ART. I. Essentials of Cooking. By J. J. Caldwell, M. D., and Geo, H Whaley, M. D., 161
ART. II. Ovariatomy by Enucleation, Report of Cases. By E. N. Brush, 164
ART. III. Foreign body removed from the Urethra. By S. A Harcourt, M. D., 170

### MISCELLANEOUS.

Clinical Notes on the Electric Cautory in Uterine Surgery. By J. Byrne, M. D., 172
Syphilis, 187
A Lesson in Diagnosis, 189
A Peculiar Case, 189
Green Wall Paper, 190
Glycogen 191
Foreign Body in Interior of Eye, 191
Case of Gastrotomy, 192
Epilepsy Cured by use of Bromide of Potassium and Sulphate of Atropia, 193
Auchylosis of the Lower Jaw, Operation, 193
Boldo, 193

### EDITORIAL DEPARTMENT.

Death of Dr. O. K. Parker, 194
Meeting of the New York State Medical Society, 195
Albany Co. Medical Society.—Medical Association of Central New York. The Medical Record, 195

BOOKS REVIEWED:

A Practical Treatise on Renal and Urinary Diseases. By Wm. Roberts, M. D., 196
Lessons in Physical Diagnosis By A. L. Loomis, M. D., 196
Diagnosis of Ovarian Tumors with special reference to the operation of Ovariotomy By W. L. Atlee, M. D., 197
A Treatise on Diseases of the Nervous System. By Wm. A. Hammond, M. D,, 197
The Anatomy aud Development of the Rodent Ulcer. By J. Collins Warren, M. D., 197
Practical Lessons in the Nature and Treatment of the affections produced by the Contagious Diseases. By John Morgan, A. M., M. D., 198
Transactions of the American Medical Association, 198
Hand Book of Compound Medicines By Arnold J. Cooley, 199
Remarks on the Stricture of the Urethra of Extreme Calibre. By F. N. Otis, M. D., 199
The Physicians Visiting List 1873, 199
A New Method of Extraction of Cataract. By R. Liebrich, 199
Books and Pamphlets Received, 200

